# How the Dynamics and Structure of Sexual Contact Networks Shape Pathogen Phylogenies

**DOI:** 10.1371/journal.pcbi.1003105

**Published:** 2013-06-20

**Authors:** Katy Robinson, Nick Fyson, Ted Cohen, Christophe Fraser, Caroline Colijn

**Affiliations:** 1 University of Bristol, Bristol, United Kingdom; 2 Imperial College London, London, United Kingdom; 3 Harvard School of Public Health, Boston, Massachussetts, United States of America; University of California San Diego, United States of America

## Abstract

The characteristics of the host contact network over which a pathogen is transmitted affect both epidemic spread and the projected effectiveness of control strategies. Given the importance of understanding these contact networks, it is unfortunate that they are very difficult to measure directly. This challenge has led to an interest in methods to infer information about host contact networks from pathogen phylogenies, because in shaping a pathogen's opportunities for reproduction, contact networks also shape pathogen evolution. Host networks influence pathogen phylogenies both directly, through governing opportunities for evolution, and indirectly by changing the prevalence and incidence. Here, we aim to separate these two effects by comparing pathogen evolution on different host networks that share similar epidemic trajectories. This approach allows use to examine the direct effects of network structure on pathogen phylogenies, largely controlling for confounding differences arising from population dynamics. We find that networks with more heterogeneous degree distributions yield pathogen phylogenies with more variable cluster numbers, smaller mean cluster sizes, shorter mean branch lengths, and somewhat higher tree imbalance than networks with relatively homogeneous degree distributions. However, in particular for dynamic networks, we find that these direct effects are relatively modest. These findings suggest that the role of the epidemic trajectory, the dynamics of the network and the inherent variability of metrics such as cluster size must each be taken into account when trying to use pathogen phylogenies to understand characteristics about the underlying host contact network.

## Author Summary

Different pathogens spread between hosts in different ways, and the nature of the resulting networks of relevant host contacts can affect the spread and control of infections. While gathering data on host contact networks is very challenging, there is growing interest in using information about the pathogens themselves to understand transmission. In particular, as genomic sequencing becomes increasingly accessible, we have the opportunity to learn more about pathogen spread and evolution by constructing pathogen phylogenetic trees (phylogenies) from gene sequences. In this paper we aim to understand how networks of contacts between hosts affect the evolution of pathogens. This matter is complicated by the fact that contact networks can have large effects on how many infections there are, and the size of pathogen populations also affects their evolution. We designed an approach to simulate pathogen transmission and mutation over host contact networks and found that accounting for both the network's effects and population dynamic effects is necessary to use phylogenetic data to understand underlying patterns of host contacts.

## Introduction

The structure of human contact networks both facilitates and constrains the spread of pathogens. Particularly for sexually transmitted infections (STIs) and infections spread via shared intravenous drug use, specific types of contact are needed for transmission to occur, and people are likely to know when a relevant contact has occurred. The realisation that heterogeneity in contact numbers can have a large impact on the transmission of a pathogen [1] has spawned considerable interest in networks in epidemiology. Research ranges from developing theoretical tools relating network structure and dynamics to the spread and control of pathogens [2]–[9] (to list but a few), and in developing sampling strategies to learn more about networks [10]–[12].

Data on human contact networks are notoriously difficult to gather 13–16. For sexual contact networks, contact tracing and snowball sampling are good approaches [17], [18] but they depend on individuals being willing to name others, which has disadvantages where the underlying behaviour is stigmatised or illegal. Respondent driven sampling (RDS) aims to address this last concern by rewarding individuals for recruiting their contacts to the study, but without requiring them to name these contacts (each individual decides whether to participate) [10]. But for many infections, such as those transmitted by casual respiratory contact, individuals will not even know who most of their contacts are. All sampling approaches used for measuring host contact networks share certain drawbacks: they access only a small portion of the population; they are sensitive to the choice of the individuals with whom the sample originates and they are not representative samples (though ideal respondent-driven sampling can give results independent of the seeding individuals [10]). In addition, they often cannot measure the dynamic aspects of contact networks, though individuals do report relationship durations in some cases [14]. Due to these constraints, survey samples may not provide sufficient characterisation of the nature of contact networks to inform network simulation models or direct public health interventions to the optimal part of the network.

As the cost of genome sequencing falls, interest in using genetic data to understand epidemic patterns has grown [19]–[27]. It is now possible to obtain sequence data from large numbers of isolates in epidemiological studies [28]–[31], [31]–[33], and the collection of these data is becoming increasingly common. Because a pathogen's opportunities for reproduction depend on the contacts made by its host, we expect host contact patterns to influence pathogen evolution. Recent work [24], [34] indicates that host contact networks can have significant effects on phylogenetic trees, and indeed, phylogenetic methods are now being used to study a range of epidemiological phenomena, particularly for viruses [35].

**Figure 1:**
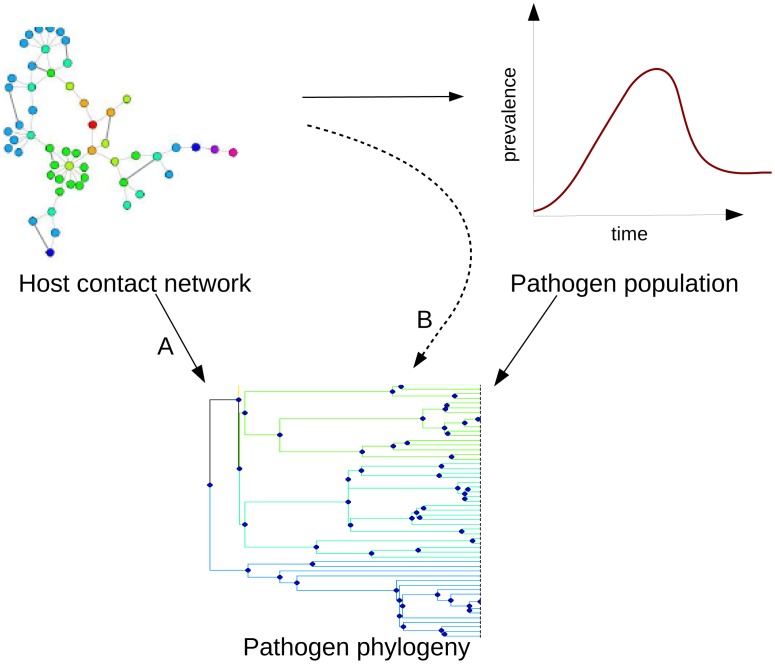
Schematic illustrating the dependence of pathogen phylogenies on both the host contact network and the pathogen's population dynamics. Route A: direct effect of the host contact network on the pathogen phylogeny. Route B: the host contact network changes the population dynamics, sometimes dramatically, and this in turn affects the pathogen phylogeny.

Pathogens with identical biological characteristics can cause very different types of epidemics depending on the structure of the underlying host contact network (see [4], [7], [36]). In networks with sufficiently long-tailed degree distributions, large epidemics can be caused even by pathogens with very low transmissibility [2], [37]. As described by both coalescent theory and birth-death models [38]–[41], these differing population dynamics affect phylogenetic trees [34], [42]. Phylogenies can be used to infer population dynamics [39], [43], [44], although recent work indicates that network effects can reduce the accuracy of some estimates [23]. We have schematically illustrated the mechanisms by which the host contact network and the pathogen population dynamics shape pathogen phylogenies (Figure 1). The route labelled ‘A’ represents direct effects of the network in influencing the pathogens' genetic diversity and patterns of ancestry, and hence the tree. The route labelled ‘B’ represents how network structure can indirectly influence diversity by modifying the population dynamics of the pathogen. This latter indirect route is where coalescent theory and population genetics are well developed.

Epidemic reporting such as incidence notification, combined in some cases with inference of prevalence and/or incidence from pathogen phylogenies themselves, means that we typically have at least some understanding of the stage of an epidemic at the time substantial data are available. But whether, *given comparable epidemic stage and history*, properties of phylogenetic trees can help us to infer otherwise unknown properties of the host contact network over which the pathogen is spreading is important. We would expect different future epidemic trends for different networks (even if the current stage looks the same), and we would aim to use different interventions based on what type of contact network exists [7].

The relationship between host contact networks and pathogen phylogenies may also be affected by the dynamic nature of host contact networks (i.e. the contacts relevant for pathogen transmission are often not fixed over time). Using static networks, Leventhal et al [24] concluded that heterogeneity in host degrees increases the imbalance of phylogenetic trees. But, particularly in the case of very heterogeneous networks, the numerous contacts of the most highly active individuals are not likely to be made at the same time. Rather, evidence for heterogeneity in contact numbers comes from studies asking for the number of contacts people have had over relatively long time periods, such as over 5 years or a lifetime [45]. The relationship between the aggregated long-term host contact network and the actual transmission network over which the pathogen is transmitted depends on the duration of infectiousness and the host contact dynamics [5]. This may affect the relationship between the contact network and pathogen phylogenies.

Here we ask to what extent dynamic host contact networks shape pathogen phylogenies, under conditions when the epidemic has similar trajectories on different networks (i.e. we examine route A in Figure 1). We use two distinct types of networks, each of which can be either static (with edges maintained over time) or dynamic (with edges changing over time). One type of network is based on the 2000 British National Survey of Sexual Attitudes and Lifestyles (‘Natsal 2000’) [14], and the other type of network is a randomly wired network based on an Erdos-Renyi (ER) random graph; the ER-type networks have considerably less heterogeneity in the numbers of contacts than the NATSAL networks. We simulate pathogen transmission and evolution over these networks, sample infected individuals and then construct phylogenetic trees from the sampled sequences. We compare the trees' branch lengths, number of clusters, and tree imbalance.

## Materials and Methods

### Network formation and dynamics

NATSAL-like dynamic contact networks were created by the method reported in [5]; further detail is provided in the supplement, Text S1. These data are publicly available through the UK Data Archive (www.data-archive.ac.uk). Erdos-Renyi style networks were created using the same general approach but using a Poisson distribution for the cumulative degree over the simulation time frame.

### Pathogen population dynamics

For each network, we tracked the number of infected hosts over time. Epidemic trajectories depend on the pathogen's duration of infectiousness and on its transmissibility. We used pathogens with durations of infectiousness of 10 weeks and 40 weeks (approximately 10 months), representing relatively short- and long-duration sexually transmitted pathogens such as *Neisseria gonorrhoeae* and *Chlamydia trachomatis*, respectively [46]. The 40 week duration results are shown in the main text; others are in the supplementary Text S1.

We then selected simulations matched by pathogen prevalence and incidence over time based on sum-of-squares differences between these curves, together with visual inspection of the time traces. Minimizing sum-of-squares differences requires weighting incidence compared to prevalence, and we found that choosing such a weighting relied on visual comparison of trajectories. While several algorithms aiming to create perfectly or very closely matched epidemics on both networks were developed, these had the drawback that they frequently returned no infection on either network (a perfect match of population dynamics, but in a trivial sense). Results reported here are based on prevalence and incidence matching based on visual inspection, and we were able to compare phylogenetic trees arising from epidemics with very similar population dynamics. For static networks, we adjusted the transmission probability to generate simulations in which the pathogen prevalences were similar despite structural differences between the network types. For the dynamic networks, tuning the duration of relationship parameter 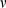 was sufficient to yield qualitatively matching simulations (Figure S1 in Text S1).

### Sequence transmission and evolution

The parameters we use are relevant for sexually transmitted pathogens with relatively long durations of infectiousness (10 or more weeks) and for which sufficient mutations occur for phylogenetic methods to be able to resolve transmissions. These requirements are met by a variety of viral and bacterial pathogens causing sexually transmitted infections, in particular chlamydia, gonorrhea and HIV. In all of these, the importance of network structure and the role of core groups in transmission is well established.

One node was randomly selected to be the source of infection, and the pathogen was then allowed to spread through the network according to its edge structure, timings and the probability of transmission. The pathogen was introduced after a ‘burn-in’ period of 50 weeks, so that results are taken from a time when the relationship dynamics were well established and the number of edges was approximately constant. The simulation strategy was that of a Gillespie simulation; the time to the next transmission event by an infected node was drawn from an exponential distribution with mean determined by the transmission rate, and the recipient node was chosen uniformly at random from the node's active contacts at that time.

The invading pathogen was assigned a randomly-generated 2500-character string of A, C, T and G, representing its genetic sequence. This string was passed, possibly with mutations, to each newly infected node. Some base positions were designated “invariant” (20%), and no mutation happened at these sites. All mutations are considered neutral. A similar approach was used by [47]. The mutation rate was an average of 3 mutations per week, to ensure that even rapid successive transmissions by the same individual were likely to be resolvable phylogenetically.

When a susceptible node was infected:

1. The number of mutations, 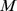, which had occurred was drawn from a Poisson distribution with mean 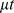, where 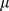 was the mutation rate and 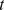 was the time since the infecting node's sequence was last checked (either for transmission or for sequence sampling). This models mutations accruing at a constant rate over time (hence the Poisson random variable and the dependence on the time elapsed). For the results in Figure S3 in Text S1 labelled ‘mutation on transmission’, an average of 3 mutations was used regardless of the time elapsed.

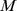
 base positions were then randomly selected from the sequence (invariant sites can not be selected) and mutated. Different rates could be used for different types of mutation, for example making transitions (A-G, C-T) more likely than transversions (A-C, A-T, G-C, G-T); the default is that all mutation types are equally likely.
3. The mutated sequence was stored as the pathogen sequence for the newly infected node.

### Homochronous sampling

At times 200 and 260 weeks, 100 infected nodes were selected at random from all infected hosts. Separate trees were constructed for each sampling time. Sampling time affects the sampling density, as the prevalence is lower earlier in the simulated outbreak. The PHYLIP (Phylogeny Inference Package) collection's DNAMLK routine was used for tree reconstruction from the sampled sequences; this is a maximum likelihood method using a molecular clock. PHYLIP is available online at http://evolution.genetics.washington.edu/phylip.html. We also explored the possibility of additional mutations occurring at the time of transmission from one host to another, reflecting the ‘bottlenecking’ used in modelling, for example, HIV evolution from one host to another [27]; results are shown in Text S1 and supplementary Figures.

### Heterochronous sampling

We used two different schemes to sample over time: uniform sampling over all infectious nodes throughout the entire simulation, and sampling at a constant rate in time throughout the simulation. These approaches differ because the prevalence of infection changed with time.

### Clustering and branch lengths

A cluster is a set of leaf nodes whose most recent common ancestor occurs at a distance less than or equal to a cut-off distance from the tree's root. In other words, if a tree is drawn with its root on the left- and leaves on the right- hand side, with branch lengths representing evolutionary distances (as determined by PHYLIP), we draw a vertical line through the tree. If the most recent common ancestor of a group of leaf nodes is on or to the right of that line, then that group of nodes makes up a cluster. Under homochronous sampling, all leaf nodes are at the same distance from the root due to the molecular clock assumption of the program used to build the trees (DNAMLK). The cut-off point used was 0.06 substitutions/site for the clustering results unless otherwise indicated; this is approximately one quarter to one third of the root-to-leaf distance for most trees. We varied this choice to ensure our results were not an artifact of the cut-off value. We also used cut-offs that were a given portion of tree's total distance (the distance to the most recent common ancestor of all leaves) (see Text S1). Branch lengths were computed by DNAMLK (DNAML for heterochronous sampling). For all trees with labels, units are in substitutions/site.

### Tree imbalance

A binary tree is considered to be perfectly balanced if each internal (branch) node in the tree divides the leaves descending from it into two equally-sized groups. The degree to which a tree diverges from the perfectly balanced tree with the same number of leaf nodes is its tree imbalance, for which several measures exist. Several of these measures have previously been found to be associated with variation in speciation rates among different strains or species [48], [49]; Agapow and Purvis [50] compared eight such measures and noted that differences in the location of tree imbalances (spread throughout the whole tree, or more concentrated towards its root) could be associated with different evolutionary processes.

One of the most widely-used measures of imbalance is Colless's I, corrected by Heard to take account of different tree sizes [51]. This was calculated here using the following equation: 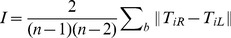 where 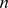 is the number of leaf nodes (number of samples), 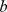 is the set of internal branch nodes and 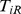 and 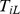 are the number of leaf nodes descending from the right and left branch of internal node 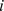 respectively. The expected value of I for a tree with n leaves [51] is given by 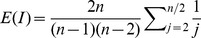 when 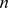 is even, or 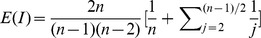 when 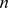 is odd.

The programs developed for network generation, dynamics and pathogen transmission, evolution and sampling were written in C++.

Box plots were created using MATLAB. In each box plot, the median is indicated in red and the 25th and 75th quantiles correspond to the limits of the boxes. Whiskers (black lines) extend to the range not considered to be outliers, and outliers are marked individually with red
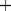
symbols.

## Results

**Figure 2:**
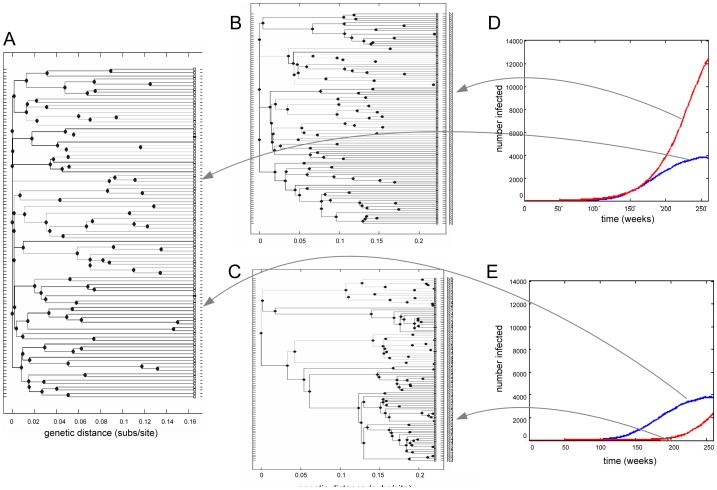
Trees constructed from pathogens spreading on the NATSAL and ER networks supporting very different epidemic trajectories. A: Phylogenetic tree from the NATSAL network, corresponding to the pathogen prevalence in panels D and E (blue lines). B and C: tree derived from the pathogen spreading on an ER network, corresponding to the red lines in D and E respectively. The ER prevalence was varied by changing the transmission parameter. Sampling was done at time 260 weeks.

Figure 2 shows trees typical of samples taken from NATSAL and ER-type networks. The NATSAL network's tree (panel A) shows relatively long branch lengths to the leaves (i.e. samples) and short branch lengths near the root. In contrast, the trees resulting from simulated evolution on the ER-type networks (panels B and C) show somewhat more uniform branching through time. However, these patterns are strongly affected by the population dynamics. Under coalescent theory, the time during which there are 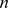 lineages is exponentially distributed with mean 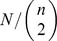 [52]. The exterior branches (those terminating in leaves) of the three trees illustrate this qualitative dependence on the population dynamics: 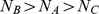, and 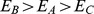 (where 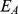 is the mean exterior branch length of tree 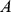, 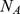 is the prevalence of infection near the sampling time of 260 weeks and similarly for 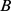 and 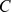). However, we observe qualitatively different tree shapes for very similar patterns of early prevalence and incidence (early portion of trees in panel A and B of Figure 2), and also for epidemics with similar prevalence at the time of sampling (panels A and C). These simulations suggest that networks influence the relationship between the number of lineages through time in a tree and the epidemic trajectory of a pathogen.

Figure 2 illustrates two points motivating the approach we take in the remainder of this work. The first is that pathogen population dynamics can have a stronger impact on tree structure than the network; the trees in panels B and C are both derived from ER networks but look quite different. The second is that network structure retains influence over pathogen phylogenies independent of population dynamics: the NATSAL-derived tree in panel A is different from the tree in panel B over the early period of the epidemic when the epidemic trajectories are similar. Disentangling these two effects is challenging, because networks play a major role in determining patterns of pathogen incidence and prevalence.

To elucidate the direct effects of the underlying network structure on phylogenetic trees (i.e. pathway A in Figure 1), we compared trees drawn from simulations in which the networks' transmissibility and the durations of contacts were adjusted to produce matched epidemic trajectories.

### Clustering

**Figure 3:**
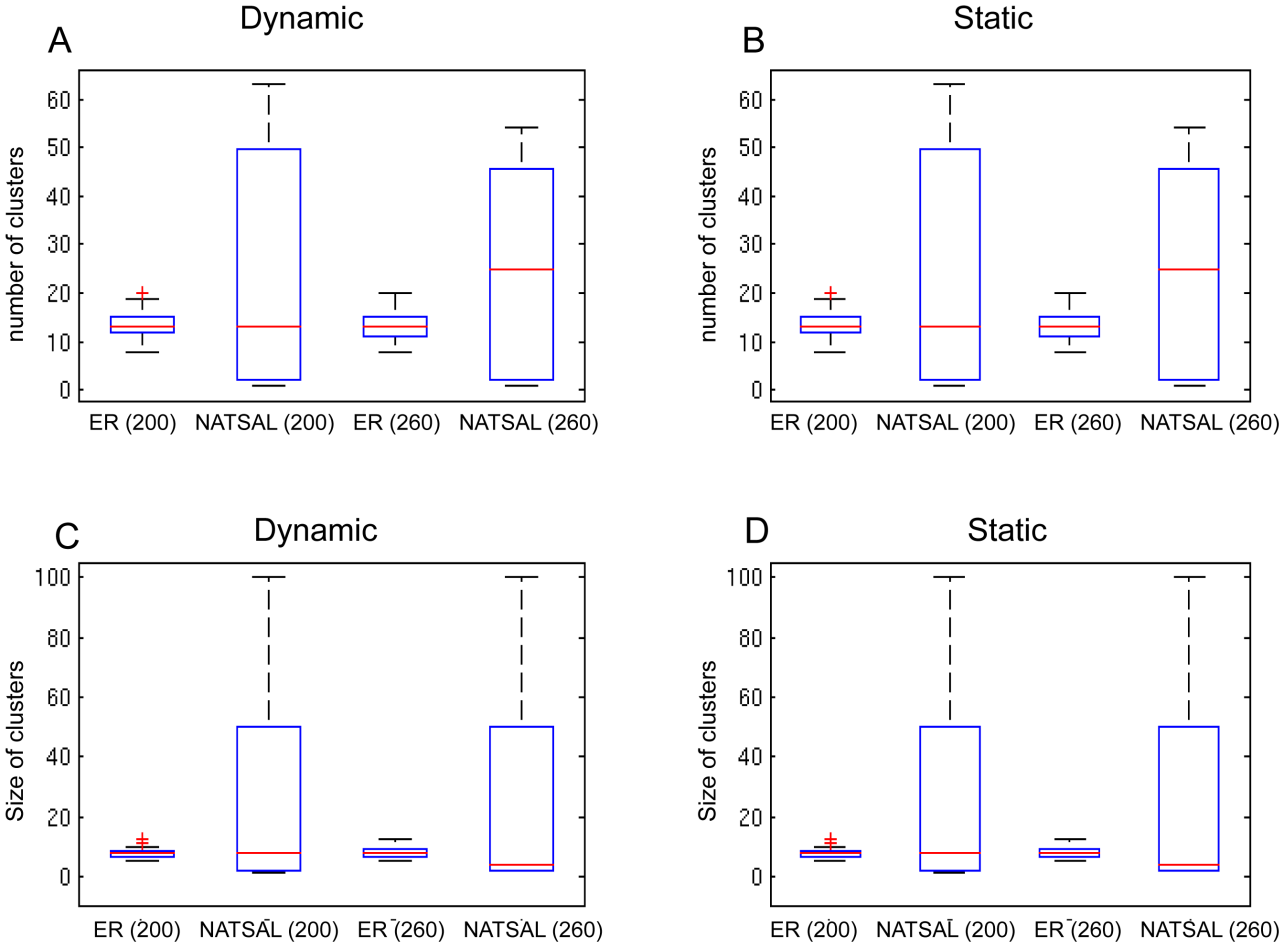
Mean number and mean sizes of clusters of two or more samples in phylogenetic trees from the scenarios. A: cluster numbers (dynamic with duration 40 weeks); B cluster numbers (static network); C cluster sizes (dynamic with duration 40 weeks); D cluster sizes (static network).

Figure 3 shows the mean numbers and mean sizes of clusters in phylogenetic trees derived from dynamic and static underlying networks with duration of infectiousness of 40 weeks. (A shorter duration, and the case where mutation occurs on transmission rather than over time are shown in the supplement, Text S1). In the boxplot figures, the boxes should each be considered as two pairs, with the first pair comparing the networks at the first sampling time (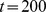), and the second pair showing tree summary statistics from the second sampling time (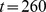). For reasons of space Erdos-Renyi networks are labelled ‘ER’.

The NATSAL-based networks have greater variability in cluster count, which is particularly evident in the static network case. Because the ER networks are more regular in structure, the pathogen spreads at a similar rate, regardless of the starting point. In contrast, the underlying heterogeneity of the NATSAL-based networks causes incidence to rise quickly when the pathogen reaches a high-activity node, which due to assortativity is likely to be linked to other high-activity nodes. We note that the distribution of cluster numbers and cluster sizes depends on the choice of cutoff; results for different cut-offs are shown in Figure S5 in Text S1. Furthermore, using a cut-off measured in substitutions per site (i.e. in the branch length units computed by DNAMLK) does not account for the fact that each tree has a different distance between its leaves and their most recent common ancestor, so that a cut-off of 0.06 may be halfway along one tree but 2/3 of the way along another. For this reason, we also computed the mean cluster number for cut-offs at fixed portions of the trees' total distance (Figure S6 in Text S1). This reduces the heterogeneity of the results derived from NATSAL networks. But both illustrate the same relationships as shown in Figure 3, namely that NATSAL networks have more clusters and their numbers and sizes are more variable. The sizes of clusters are generally inversely related to the number of clusters (as the numbers in total must add to the number of leaves).

Even under conditions when the overall prevalence and incidence are similar in the two networks, the NATSAL networks' transmission patterns are likely to involve a small number of high-activity individuals, whose descendant infections will be related and form clusters in the phylogenetic trees. Variation in cluster numbers arises due to the interplay between when high-degree individuals are reached by the pathogen, when their descendant infections arise (and hence how correlated these are genetically), and the extent to which these descendants are sampled. This effect is not a consequence of population dynamics but is directly related to network structure.

**Figure 4:**
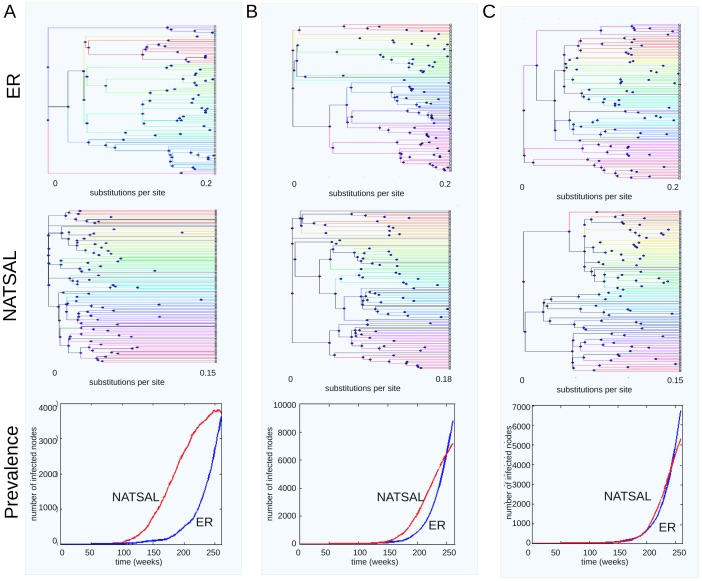
Comparison of typical trees derived from ER-like networks (top row) and NATSAL networks (middle row) illustrating that pathogen prevalence (bottom row) as well as networks both influence trees. The NATSAL trees displays early divergence compared to the ER trees, and this affects the number of clusters. Panel A shows different epidemic trajectories and their corresponding trees, B shows more similar trajectories, and C shows closely matched epidemics. The tree differences are most modest in panel C where the pathogen population dynamics are closely matched. Edges in each cluster are drawn with the same colour. The threshold value for clustering was 0.1.

Differences in clustering patterns between NATSAL and ER networks are also apparent by visual comparison of the trees shown in Figure 4, which depict the distinct clusters with different edge colours. In panel A, the NATSAL-based tree is divided into 34 small clusters (the largest of which contains 6 samples), while the ER-based tree has only 7 clusters which are more varied in size, containing 2, 2, 5, 13, 17, 17 and 21 nodes. Panel B demonstrates the same qualitative effect, in which the NATSAL network gives rise to more clusters than the ER network. In panel C, where the entire epidemic trajectory matches most closely, the difference remains but is more modest. Cluster size is related to the inferred times of the earliest internal nodes: because cluster numbers depend on a cut-off value, trees with earlier nodes have more clusters for the same cut-off value.

**Figure 5:**
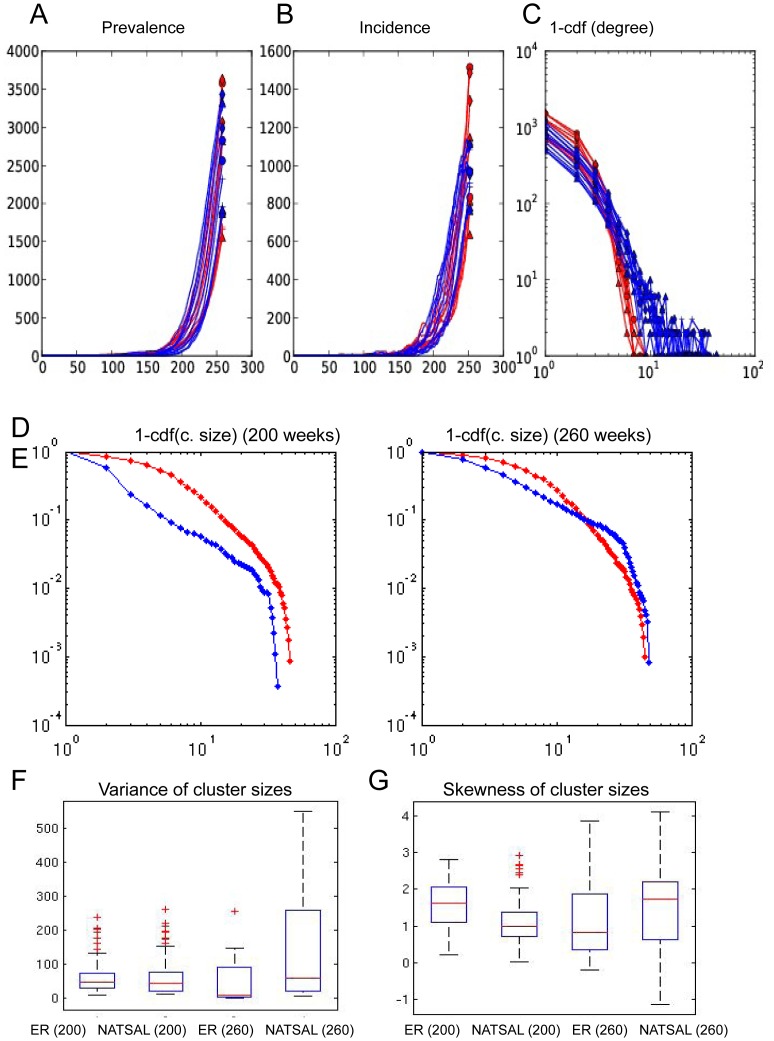
Network prevalence, incidence and 1-cumulative degree distributions for ER (red) and NATSAL (blue) dynamic networks with 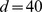. Note that the NATSAL network admits similar epidemic trajectories with markedly different degree distributions (A–C). Panels D, E show the cumulative distributions of the cluster sizes in the ER (red) and NATSAL (blue) networks, and illustrate that these do not parallel the degree distribution; NATSAL networks do not have particularly more variable cluster sizes within trees. Panels F and G show the variance and skewness in boxplots; each box represents all trees from the given network and time point as in other figures.

Past work has implicitly assumed that clustering patterns in trees would mirror the underlying contact network's degree distribution [53]–[56], though more recent work notes that clustering patterns do not necessarily reflect network strucure [27], [57]. In Figure 5, we show the distribution of cluster sizes in phylogenetic trees and the degree distribution of the transmission trees on which infection spread. We find that that the cluster size distribution does not particularly mirror the underlying contact network's degree distribution: while the NATSAL networks have a much broader degree distribution, both in general and among those actually infected even when prevalence and incidence match well, the distributions of cluster sizes within the trees are very similar. We computed the variance and skewness of the cluster size distribution within each tree, and these were also not particularly different between the networks.

### Branch lengths

**Figure 6:**
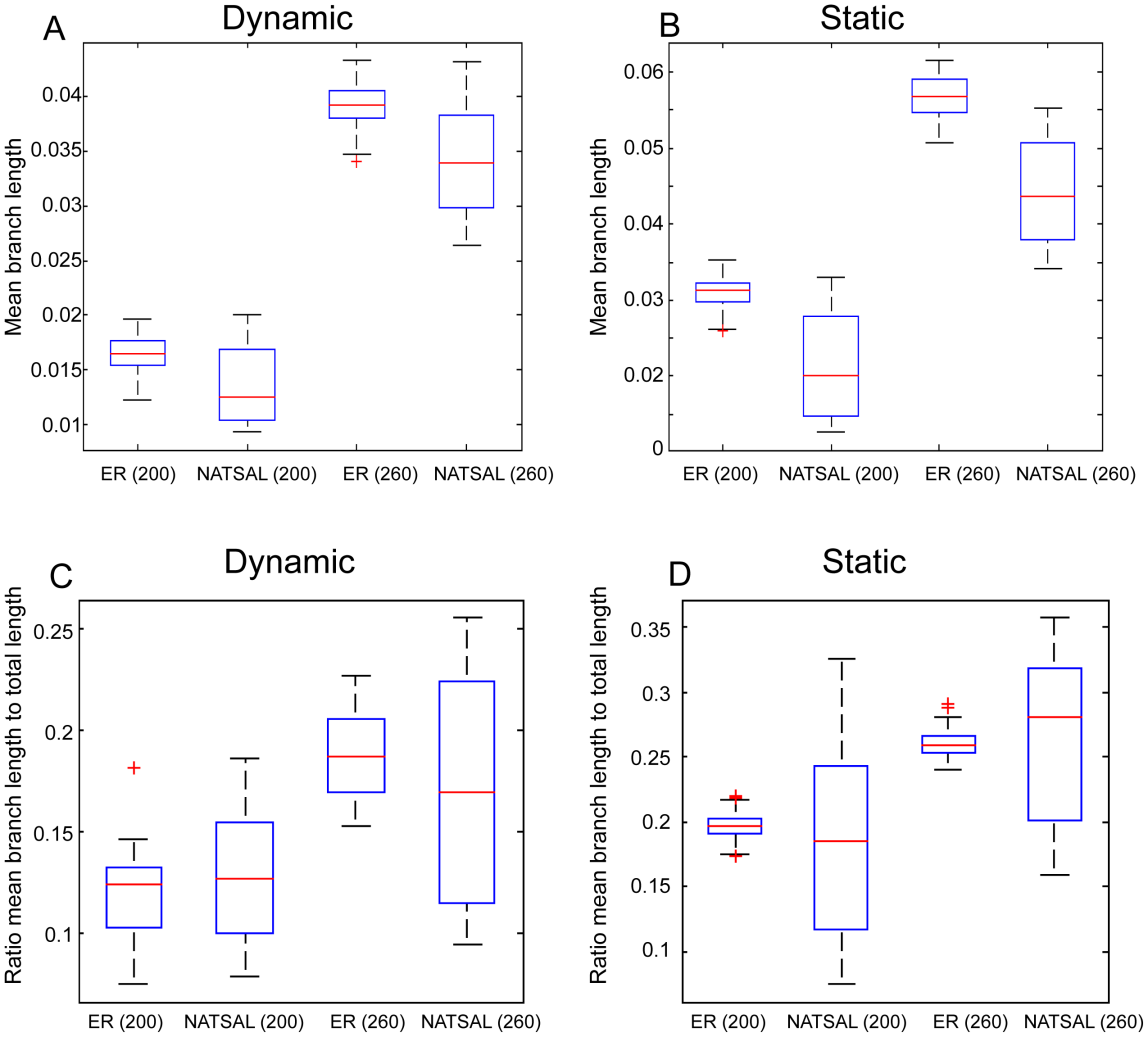
Branch lengths in phylogenetic trees from the scenarios. A: mean branch lengths in trees from dynamic networks with a pathogen with duration of infectiousness 40 weeks; B mean branch lengths in static networks with duration of infectiousness 40 weeks. C, D: ratio of mean branch length to total tree distance, from dynamic and static networks.

We compared the mean branch lengths of the phylogenetic trees arising from pathogens sampled from epidemics spreading on the different networks (see Figure 6). A significant difference in the mean branch lengths was found between the ER and the NATSAL-based groups at both sampling times, with ER networks producing trees with higher mean branch lengths, particularly in the static network case.

Differences in mean branch lengths may reflect either variation in the root-to-leaf distance from one tree to another, or differences in where the longer branches are (with short branches close to the root requiring long branches closer to the leaves, where there are more branches, resulting in a higher mean branch length). Both are at play here. We find that the root-to-leaf lengths are generally shorter in the NATSAL network-derived trees, probably because of relatively shorter transmission routes (shorter in time as well as smaller in numbers of edges traversed). A higher portion of transmission in heterogeneous networks stems from a small number of high-activity nodes, accounting for these shorter paths. In this way, the most recent common ancestor of the sampled sequences may not in fact be the common ancestor introduced in the simulation, but some subsequent infection that reached a high-degree individual. This occurs more frequently in NATSAL-derived trees than in the ER-like networks. We also find that the ratio of mean branch length to total branch length is far more variable in trees derived from NATSAL networks than it is in those derived from ER networks. While the differences in mean branch lengths are due to differences in the total genetic distance in the trees, the differences in variability are due to more variable allocation of the trees' total distance between early and later branches.

**Figure 7:**
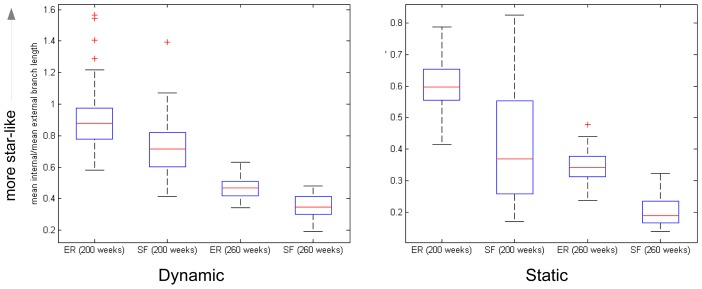
Mean internal/external branch lengths for trees derived from epidemics on dynamic (d = 40 weeks) and static networks.

We also compared internal and external (pendant) branch lengths in the trees. A high ratio of internal branch length to external branch length occurs in ‘star-like’ trees. Volz found that faster-than-exponential growth in prevalence creates less star-like trees than exponential growth [26]. We find that NATSAL-derived trees are less ‘star-like’ than ER-derived trees, as shown in Figure 7. This is consistent with Volz's finding and the shape of the prevalence curves in Figure S1 in Text S1, though this consistency does not imply that the pathogen prevalence (or prevalence and incidence) are the only factors affecting the branch lengths and ‘star-like’ quality of the trees.

### Imbalance

**Figure 8:**
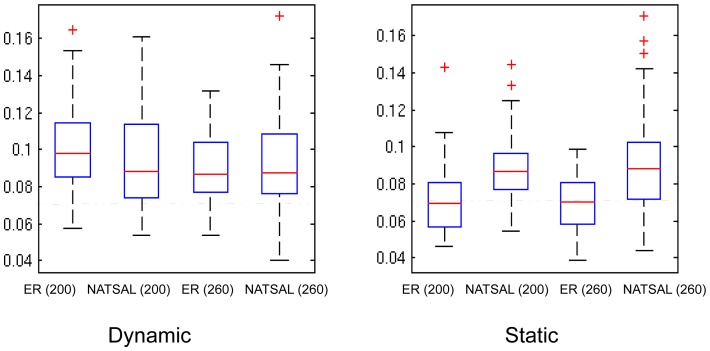
Tree imbalance in phylogenetic trees from the scenarios (left) dynamic with duration 40 weeks; (right) static networks. Dashed lines indicate the expected imbalance for trees of this size [51].

We hypothesized that the greater heterogeneity in contact numbers in the NATSAL networks compared to the ER networks would affect the structure of the phylogenetic trees. Imbalance is one way to measure tree structure, and this metric has the advantage that it is not directly affected by population dynamics (as described by coalescent theory). Figure 8 illustrates the patterns and variability in tree imbalance. We found that static NATSAL-type networks lead to greater imbalance than ER-type networks, but this difference is diminished when the networks are dynamic.

Heard [48] described variability in speciation and extinction rates as an important cause of imbalance in phylogenetic trees. Positive selection can produce tree imbalance since lineages undergoing positive selection will experience higher speciation (here, branching) rates than other lineages. Here, imbalance arises in the absense of any positive selection, but asymmetric branching rates may still account mechanistically for higher imbalance. This may help to explain the static network results, in which the ER networks produced the most balanced trees: in static ER networks, every node has approximately the same number of contacts at all time points, and so the rate of spread of infection should be relatively regular, resulting in more symmetric lineage branching rates than those in NATSAL networks where some lineages have access to highly active nodes. This finding is consistent with the recent imbalance results of Leventhal et al [24]. In contrast to the static case, we found little difference in imbalance when the networks were dynamic, though we note the difference in imbalance was more pronounced for pathogens with shorter duration of infectiousness (see Text S1).

### Prevalence and incidence matching

**Figure 9:**
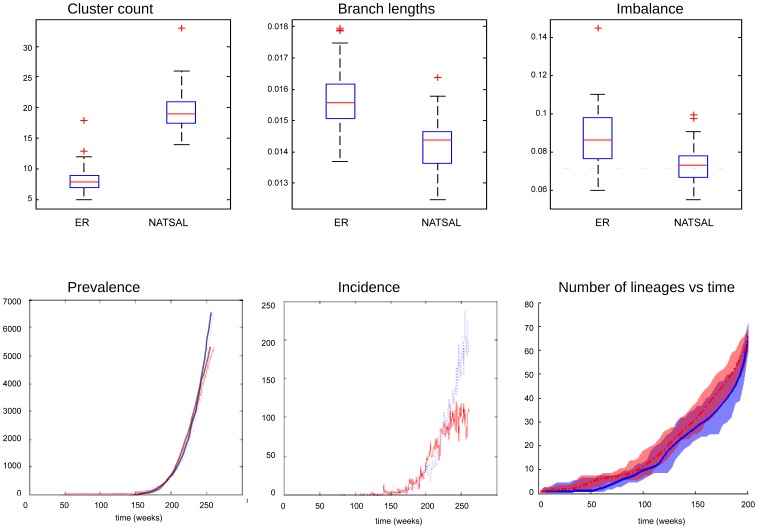
Cluster count, branch length and imbalance (top row) for a pathogen with duration of infectiousness d = 40, taken from simulations in which incidence and prevalence were as closely matched as possible. Dashed line indicates the expected imbalance for trees of this size [51]. Prevalence and incidence over time in an ER network (blue) and NATSAL-based network (red) are shown in the bottom row for dynamic ER and NATSAL underlying contact networks. The number of lineages through time (LTT) in the trees for ER (solid) and NATSAL (dotted) is also shown. The LTT plots show the LTT for all trees; mean LTT at each time are indicated with dotted and solid lines and the coloured regions range from the minimum to the maximum. Distributions were close to uniform over this range. The ranges almost entirely overlap.

Several of the comparative results presented could result from differences in incidence and prevalence. For example, NATSAL networks generate more variable population dynamics than ER networks (see Figure S1 in Text S1), and this could explain the higher variability of trees derived from NATSAL networks. To investigate the independent effect of network structure (i.e. mechanism A in Figure 1), we examined phylogenetic trees from pairs of simulations on dynamic networks in which the pathogen prevalence and incidence were very closely matched up to the 200-week sampling time (see Figure 9). To verify that this matching process does not simply select simulations for which few or no highly active individuals were infected, we found the cumulative distribution function for the degrees of those infected (Figure 5, top row); NATSAL networks consistently include individuals with 10 times as many contacts as ER networks.

Using these matched epidemics, we found similar overall patterns for branch length and clustering as we previously described: ER-derived trees have smaller cluster count and longer mean branch lengths. The NATSAL-derived trees showed somewhat lower imbalance than those from the ER networks. This finding is different than the results of Leventhal et al [24], and suggests that allowing networks to be dynamic erodes the networks' effects on imbalance.

Figure 9 shows the number of lineages in the trees as a function of the distance from the trees' roots (known as a lineages through time or LTT plot). These are similar for both sets of trees, which is to be expected since both prevalence and incidence were similar throughout the simulation prior to time t = 200 weeks where sampling was done. The fact that noticeable differences in the trees persist when the population dynamics and LTT plots are so similar indicates that the differences we have observed are not an artefact of the networks' effects on the pathogen population dynamics.

**Figure 10:**
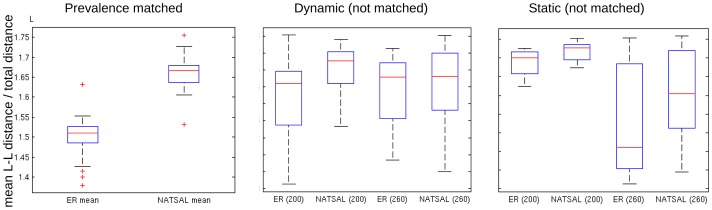
Mean leaf-to-leaf distance scaled to the total distance in each tree, for the matched prevalence scenario, unmatched dynamic ( 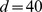
 **) and static.**

Interestingly, in examining the relationships among the branch lengths, we found that the leaf-leaf distances were different between ER and NATSAL-derived trees only when the prevalence and incidence closely matched. This is illustrated in Figure 10, which shows the leaf-to-leaf distances (scaled to the total distance in each tree, to avoid confounding the issue with the differences in total variation). Longer leaf-leaf distances in NATSAL networks reflect relatively earlier diversification along the tree. The fact that longer leaf-leaf distances only become apparent in the scenario where prevalence and incidence are closely matched is interesting; it is possible that a knowledge of the true prevalence and the leaf-leaf distances predicted under a null model could form part a strategy to characterise network structure from phylogenies. However, our results here suggest that branch lengths are not sufficient to do this.

### Heterochronous sampling

We hypothesized that homochronous sampling may be less sensitive to differences in the networks because highly active individuals are likely to be infected early, and so are unlikely to be sampled directly in homochronous sampling performed when a large outbreak has reached high prevalence. For these reasons, we repeated our analysis with two different models of heterochronous sampling: sampling each infected node throughout the simulation with uniform probability, and sampling at a constant rate in time throughout the simulation. The latter leads to a relative oversampling of nodes infected early in the outbreak, due to the lower prevalence at earlier times.

**Figure 11:**
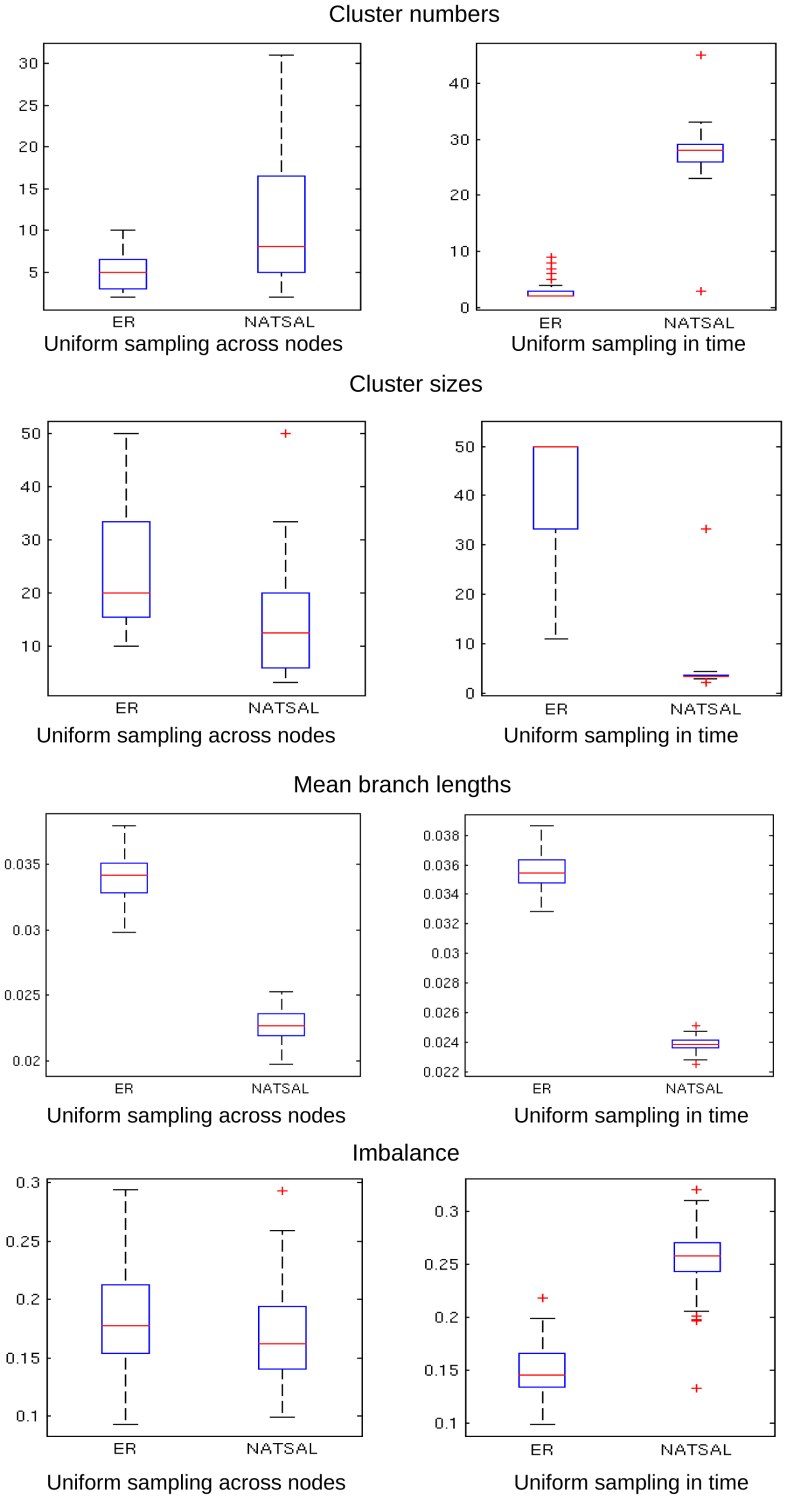
Clustering, branch lengths and imbalance for heterochronous sampling on the dynamic network with 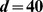. Clustering was done with a cut-off distance of 0.06 as in the results for homochronous sampling. The expected value of imbalance is 0.074 [51], considerably less than the imbalance of the heterochronously sampled trees from both networks.

The cluster numbers and sizes, branch lengths and imbalances in heterochronous trees is shown in Figure 11. As in Figure 3, trees derived from NATSAL networks have more clusters at a given cut-off, and this is particularly marked when sampling uniformly in time (so over-sampling early infections). As in Figure 6, mean branch lengths are lower in trees derived from NATSAL networks, whatever the sampling scheme. Imbalance is only different under uniform sampling in time, where we recover the result of Leventhal et al [24] that imbalance is higher in skewed networks.

## Discussion

The effect of host contact network structure on the diversification of pathogens is mediated through a complex set of interactions that involve both how networks support different epidemic dynamics, and how they present different opportunities for pathogen evolution. Understanding the independent impact of network effects on observed pathogen phylogenies is challenging. Our models suggest that the structure of contact networks affects pathogen diversification even when epidemic dynamics are similar, evolution is by neutral mutation only, and the host contact network is randomly wired apart from its heterogeneity in contact numbers. In contrast to the work presented in [24], however, we find that these effects are relatively modest, and in particular, when networks are dynamic and when epidemic dynamics are similar, networks with long-tailed degree distributions (here NATSAL-type networks) do not necessarily lead to much higher levels of tree imbalance than homogeneous networks (i.e. ER-type networks). The main exception is under a heterochronous sampling scheme in which nodes infected early are very likely to be sampled; in this case, tree imbalance is consistently higher for phylogenetic trees derived from NATSAL networks.

Much of the work to date on relating transmission patterns to phylogenies has been done on HIV [27], [53], [54], [56]; the mutation rate is high and phylogenetic methods have been in use for decades. Furthermore the duration of infectiousness with HIV, even in the initial stage, is in the appropriate range for our model. (Estimates of HIV transmission rates during different disease stages identified a period of high infectivity lasting three months [58], followed by a reduction in transmission rate until the acute phase of infection.). Bacterial STIs also have long infectious durations, with estimates of 55 days for gonorrhea [46], [59] and 10 months for chlamydia [46], [60]. While bacterial and viral pathogens have very different *per site* mutation rates, the greater length of bacterial genomes compensates for bacterias' lower mutation rates. A number of recent studies have used whole genome sequencing to resolve bacterial transmission [28], [29]. In any case, as long as the mutation rate is high enough that phylogenetic error is low, true phylogenies do not depend on the mutation rate. Here, we chose a mutation rate high enough that transmission events are highly likely to carry mutations with them.

We have designed our study to examine the effect of host network structure on pathogen phylogenies in the absence of strong differences in pathogen population dynamics, because the latter can dominate the effects on the phylogenetic trees. However, this choice has imposed a strong constraint relative to what might be known in many realistic settings, and it reduces the apparent effect of network structure. Furthermore, there is more work to be done in characterising the differing roles of population dynamics and host behaviour in shaping pathogen phylogenies. This might include fixing population dynamics and explicitly exploring re-wiring transmission trees that fit the (fixed) population trajectories, or using optimisation methods to obtain best-fit population dynamics matches over large numbers of simulations and varying network parameters. In addition, future work could use substitution processes tuned to represent particular pathogens, to generate simulated phylogenies whose properties could be compared with phylogenies derived from data. Ultimately, using phylogenetic data to detect the nature of, and changes in, host contact networks would be a worthy goal, though our results here suggest that this will require incorporating additional epidemiological information and improved summary statistics for phylogenies.

Monitoring the early emergence of a pathogen or the beginning of an outbreak is challenging, whereas more data are usually available about recent and current incidence and prevalence. Our results suggest that one way in which large sets of pathogen sequences could help inform epidemic dynamics is by shedding light on the shape of the early portion of an outbreak that may have been difficult to observe.

In some cases sequence data have been combined with other epidemiological data (contact tracing, interviews, etc.) to great effect in characterising transmission routes [29], [57], [61]. However, in many settings additional epidemiological data may be much harder to obtain than pathogen gene sequences, particularly for larger outbreaks and epidemics. Furthermore, while pathogen phylogenies may provide detailed information about the transmission trees among individuals who have already been infected and subsequently sampled, how these phylogenies can inform a broader understanding of the features of transmission networks remains unanswered. While we have use simple sampling schemes, the actual sampling approach (e.g. RDS, contact tracing, convenience cross-sectional sampling) will affect pathogen phylogenies. This information will also need to be accounted for when using phylogenetic data to make inference about transmission patterns.

Here, we found that ER-type networks can result in more variable cluster sizes despite having less variability in contact numbers than NATSAL networks. This runs counter to the intuition that cluster size and variability parallels the size and variability of the underlying contact network. The extent to which sequences occur in clusters, and cluster sizes, are a frequently reported aspect of phylogenetic data [62]–[64]. It has been implicitly or explicitly assumed that a clade in a phylogenetic tree corresponds to a transmission cluster [53]–[56], and while sequences that cluster together are related, we have found that the cluster size distribution does not mirror the underlying contact network's degree distribution. This distinction between transmission trees and phylogenetic trees has been clearly articulated by Jombart et al [57]. In our simulations, clustering metrics show high variability from tree to tree when trees are made from 100 randomly sampled individuals from the same pathogen spreading on the same network. If such high variability occurs in such idealised simulated conditions, it indicates that inferences from these patterns should be treated with caution, particularly when underlying contact networks may have long-tailed degree distributions in which variability is expected to be substantially greater.

The host contact network not only shapes the pathogen phylogenetic tree, but more fundamentally, it shapes the pathogen's opportunities for reproduction and consequently for evolution. In our results, the NATSAL networks (i.e. sexual contact networks) yield phylogenies with lower branch lengths overall, and also with proportionately early diversification, compared to more homogeneously connected ER networks (Figure 10). Early diversification can be a good strategy from the point of view of a pathogen, because numerous opportunities for advantageous evolution occur in a very short window of time (giving the host population little time to respond). However, this advantage would be greatly reduced if the infection conferred immunity, because highly connected individuals would become immune quickly, diminishing the advantage for the pathogen (our models assume a susceptible-infected-susceptible structure).

Our results support several findings described by Leventhal et al [24], including that for static networks, tree imbalance is more marked for pathogens spreading on heterogeneous networks. However, we found this effect is eroded when the networks are allowed to be dynamic and when the pathogen population dynamics are similar. None of the metrics we examined are robust detectors of network differences in the context of similar pathogen population dynamics. Comparable pathogen population dynamics imply that the epidemics would appear similar from a surveillance perspective. Accordingly, while we found network-based differences for several tree metrics, we believe that these differences are too small to robustly identify the structure of underlying host contact networks, and methods to incorporate phylogenetic and epidemiological data will need to be developed. Furthermore, if phylogenetic methods are to be helpful in characterising sexual contact networks, our results indicate that (over-) sampling nodes as early as possible is likely to be more informative than sampling later.

## Supporting Information

Text S1 Supplementary information including supplementary figures and table.
(PDF)
