# Supplementary material for: How the Dynamics and Structure of Sexual Contact Networks Shape Pathogen Phylogenies
Source: PLoS Comput Biol. 2013 Jun 20;9(6):e1003105. doi: 10.1371/journal.pcbi.1003105 (PMC3688487; doi:10.1371/journal.pcbi.1003105)
Supplement: Text S1 — Supplementary information including supplementary figures and table. (PDF) [file pcbi.1003105.s001.pdf]

Text S1

Supplementary Methods

NATSAL-based network formation

We use survey data on number of sexual partnerships over five years to create dynamic contact networks with assortative mixing by activity level, and in which individuals with higher activity levels have shorter partnerships. Edges are created by the following method: 1. Each node (i) is assigned an "aggregate degree" k_i, representing the number of contacts the node will have over the five year simulation period. The (long-tailed) distribution of k_i values is taken from data from the 2000 National Survey of Sexual Attitudes and Lifestyles (NATSAL) survey, collected in Britain from adults aged 16-44. These data are publicly available at the UK Data Archive (www.data-archive.ac.uk) [1].

2. Next, half the nodes are designated "relationship initiators" and each of these nodes (j) is given k_j start times for its attempted relationships. Start times are selected uniformly within the simulation run-time. In order of increasing start time, each initiator attempts to make links to non-initiators n who have not yet formed their desired contacts, or to other initiators if creating an edge representing a homosexual partnership. To incorporate assortative mixing, there is a 70% probability that the end-node is required to have a % similar (within 15%) k_i value to that of the start-node.

3. To model the notion that highly active individuals probably have shorter partnerships than individuals with few relationships, edge duration is chosen depending on the aggregate degree, or overall activity level, of the individuals that it links. Specifically, each node has a preferred relationship duration di, which is exponentially distributed with a mean inversely proportional to the node's degree, i.e. $d_i = M/(\nu * k_i)$ where M is the simulation time. The duration d_{ij} of an edge between nodes i and j is a weighted average of the preferred duration di of node i, and d_j of node j: $d_{ij} = d_i/4 + 3 * d_j/4$, where without loss of generality $d_i < d_j$.

This method leads to the creation of a dynamic contact network whose aggregate contact distribution matches the survey data. We use networks of 50,000 individuals and a simulation period of 260 weeks. Table S1 lists properties of the networks and Figure S1 plots the distribution of aggregate degrees over the simulation period.

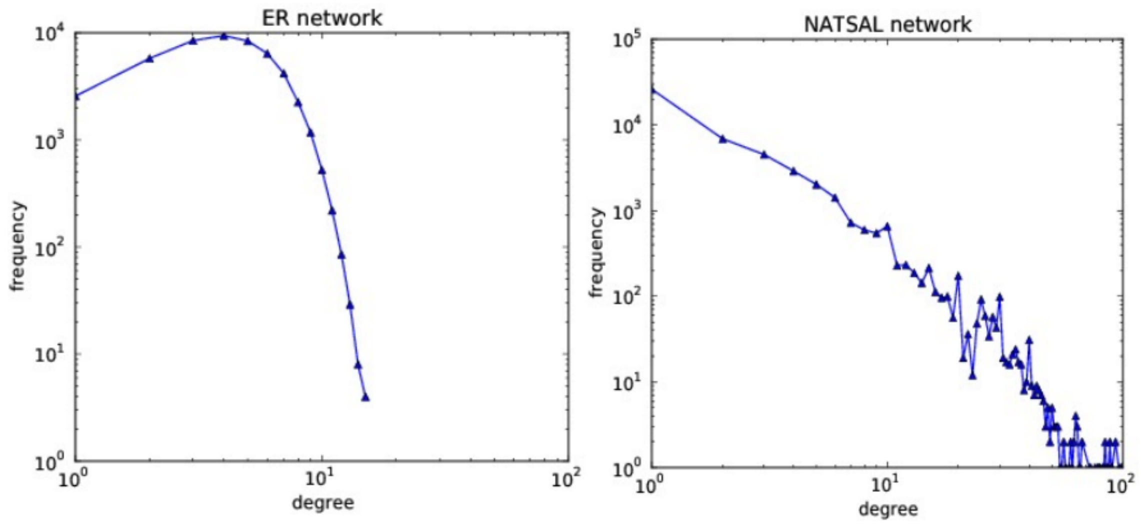

Figure S1: Degree distributions for ER and NATSAL networks

Homogeneous network formation

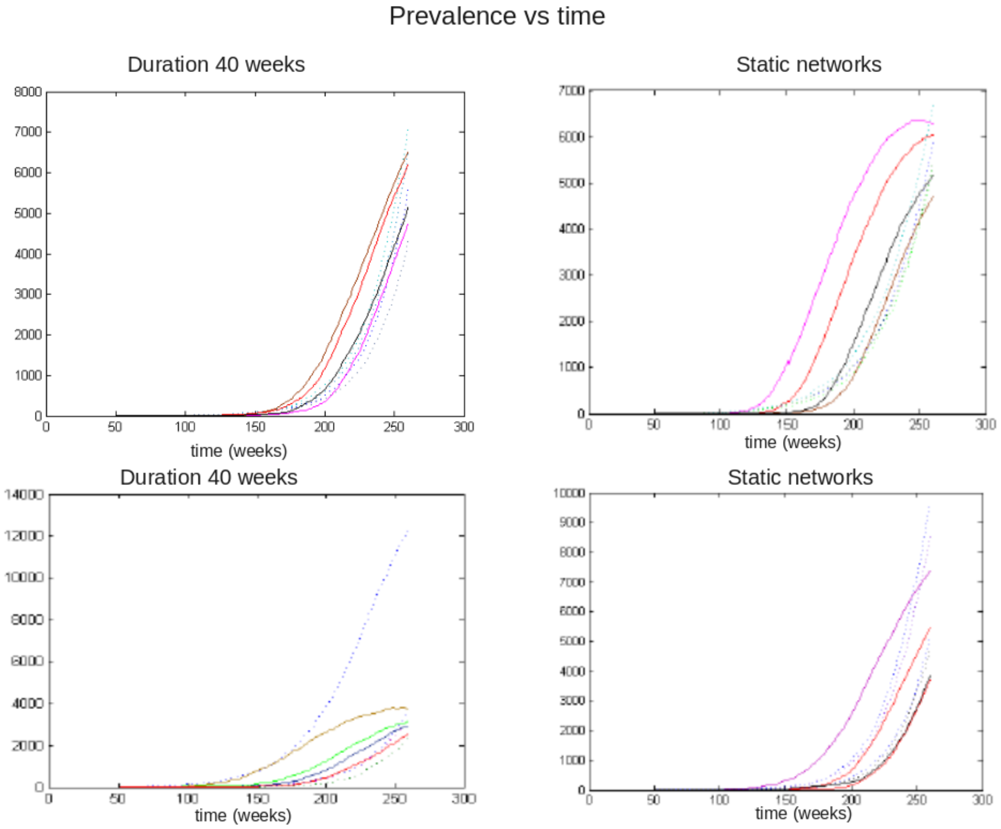

Figure S2: Prevalences for all simulations. Each plot shows the number of infected nodes as a function of time. Dotted lines correspond to NATSAL networks; solid to ER networks.

The networks produced by the NATSAL-based method show a large amount of degree heterogeneity, with contact numbers ranging from 1 to 150 over five years. In order to compare trees produced by sampling populations with different levels of contact heterogeneity, we also produced networks much more homogeneous degree distributions. These networks are formed by considering every valid pair of nodes, and (independently of all other node pairs) creating an edge between them according to a given probability p (resulting in a Poisson distribution of degree). We chose p such that the same duration of infectiousness and transmissibility resulted in broadly comparable epidemic trajectories on the static ER and NATSAL networks ($p = 0.0001$, giving a mean degree of approximately 2.25; see Table S1).

Edge duration in the homogeneous networks is calculated depending on the nodes' current degrees at the time the edge is created, as nodes are not given a target degree in this case. The same equation, $d_i = M/(\nu * k_i)$, is then used with k representing actual degree instead of target degree and ν being a constant input parameter. However, using the same value for the duration parameter ν in both networks results in the homogeneous network having a significantly higher mean edge duration, higher concurrency and consequently a larger epidemic. We compensated for this by increasing ν to 3.5.

Static networks of each type were created by discarding all information on edge timing, with all edges being active throughout the entire simulation run-time.

Supplementary Results

Figure S1 shows the prevalence of the pathogen for the simulations from which trees were derived. The top row shows the prevalence for the results given in the main text and illustrates the increased variability

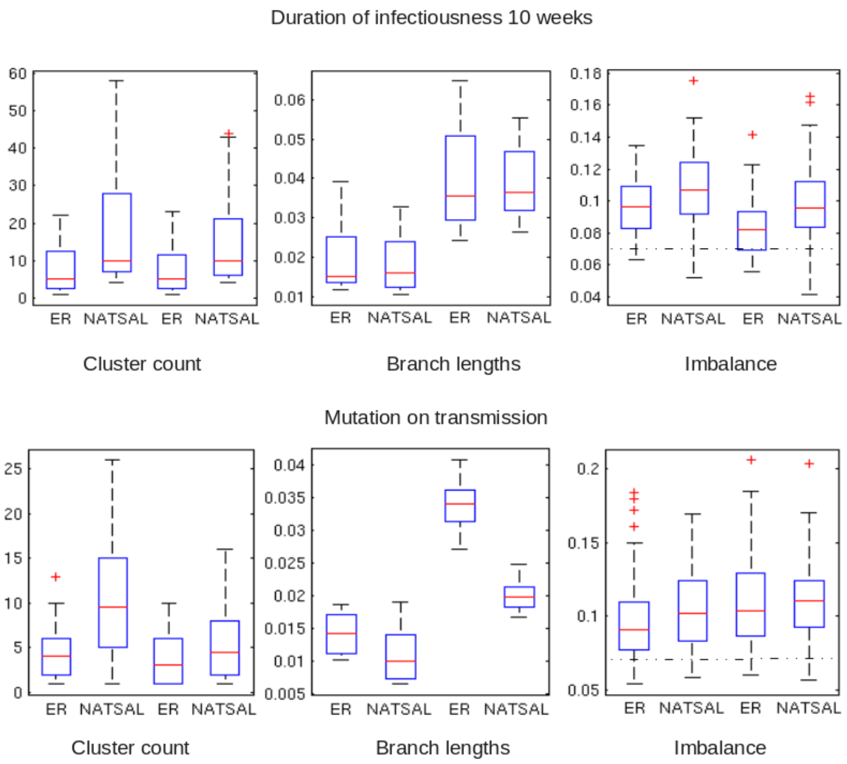

Figure S3: Cluster count, branch length and imbalance for a pathogen with reduced duration d=10 weeks (top row) and when mutation happens at the time of transmission rather than over time in hosts (bottom row). Dashed lines indicate the expected value of the imbalance, as in the main text.

in population dynamics for static networks. Transmission was curtailed on static networks to prevent a very rapid explosion in pathogen population.

We also compared trees resulting from the same networks but with a reduced pathogen duration of infectiousness of 10 weeks. The results were broadly similar to those reported in the main text, except that here we did observe a difference in tree imbalance, with NATSAL-like networks having higher imbalance in the trees than ER networks. However, the branch lengths were not appreciably different (see Figures S3 and S4).

In addition, we explored the effect of 'bottlenecking' of mutations at the time of transmission. This affects the number of mutations in a sequence passed to a new host. Again, our central results are not affected: in NATSAL-derived trees, there are more clusters, smaller branch lengths and slightly higher imbalance (see Figure S3).

Clustering

The numbers and sizes of phylogenetic clusters depend on how clusters are defined and on where the cut-off is located. For this reason, we determined the numbers and sizes of clusters in the trees for cut-offs varying between 0 and 0.1 substitutions per site, and for cut-offs at particular portions of each tree's total genetic distance. Results are shown in Figures S5, S6, S7 and S8.

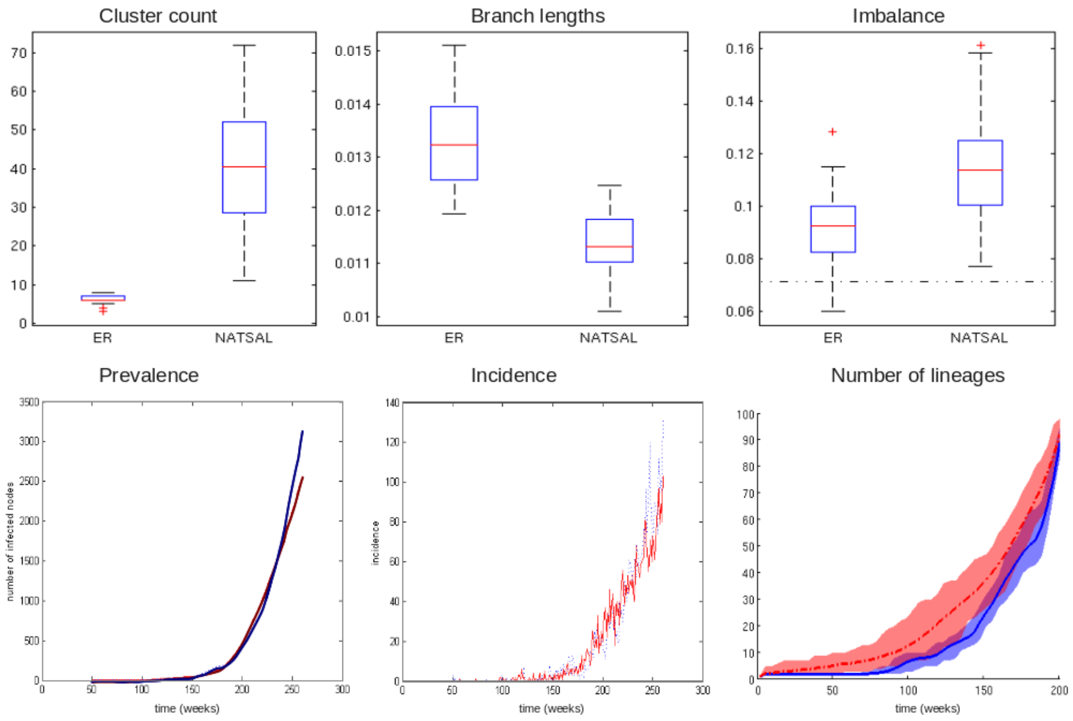

Figure S4: Cluster count, branch lengths and imbalance for prevalence and incidence matched pair of simulations with duration of infectiousness 10 weeks. Dashed lines indicate the expected value of the imbalance, as in the main text.

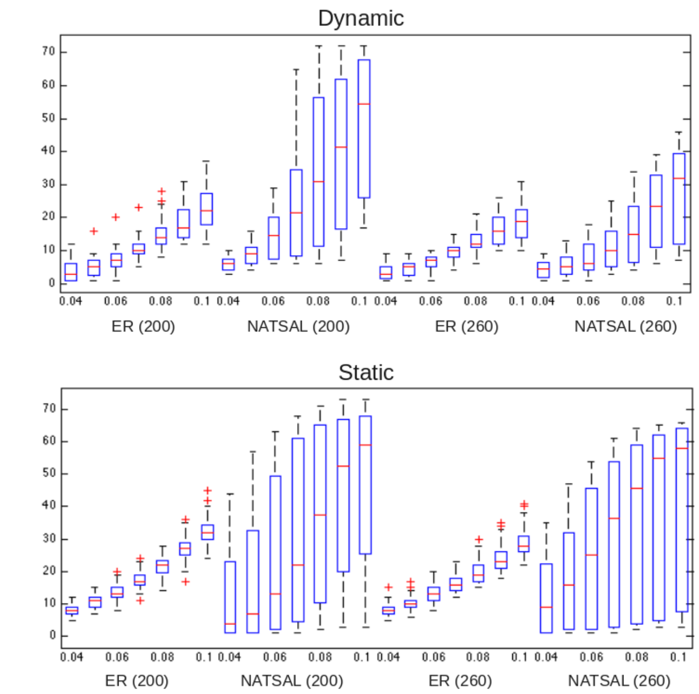

Figure S5: Numbers of clusters for varying clustering cut-off. As before, NATSAL networks yield phylogenies with more variable cluster numbers than ER networks.

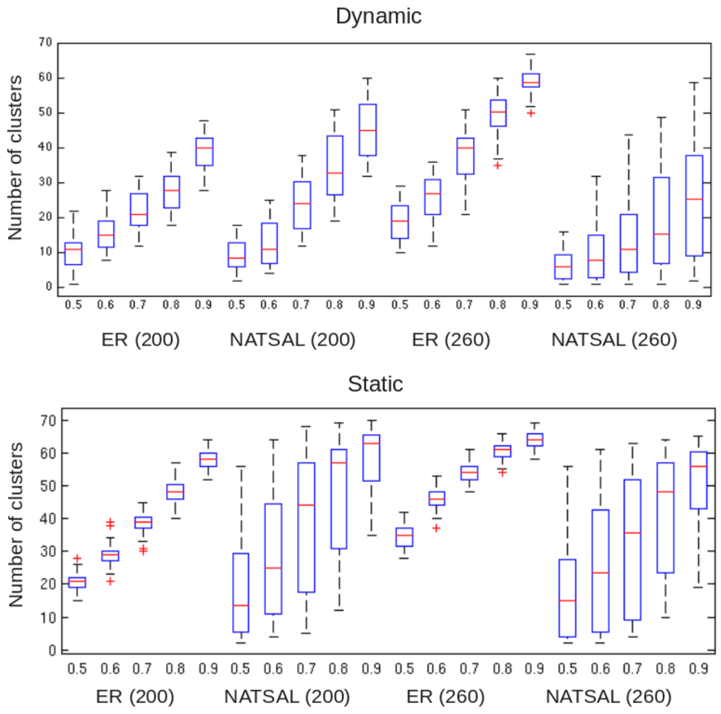

Figure S6: Numbers of clusters for varying clustering cut-off, when the cut-off is a portion of the trees' total genetic distance from leaves to root.

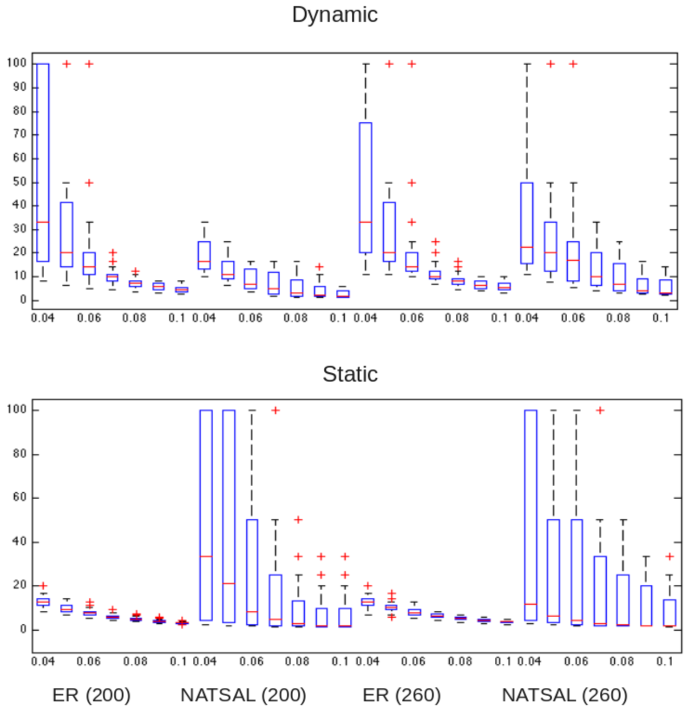

Figure S7: Sizes of clusters for varying clustering cut-off

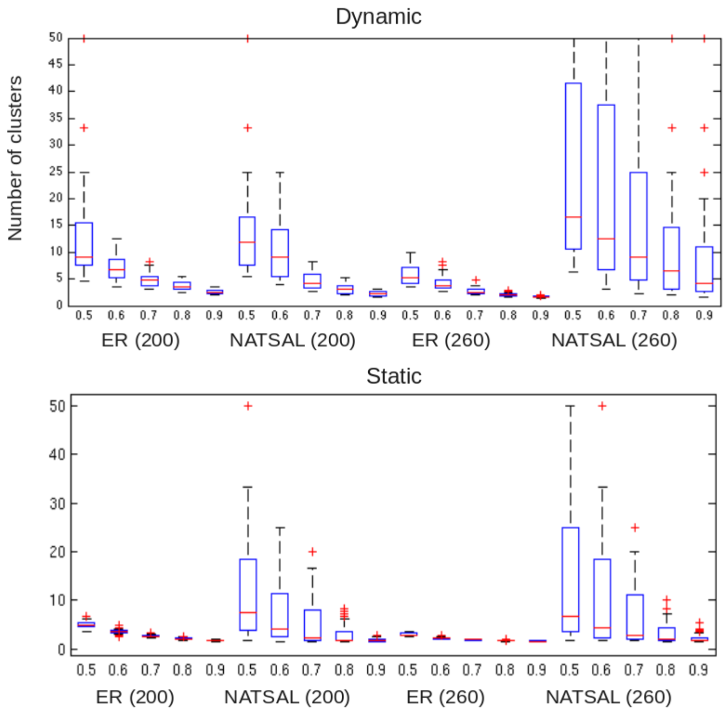

Figure S8: Sizes of clusters for varying clustering cut-off, when the cut-off is a portion of the trees' total genetic distance from leaves to root.

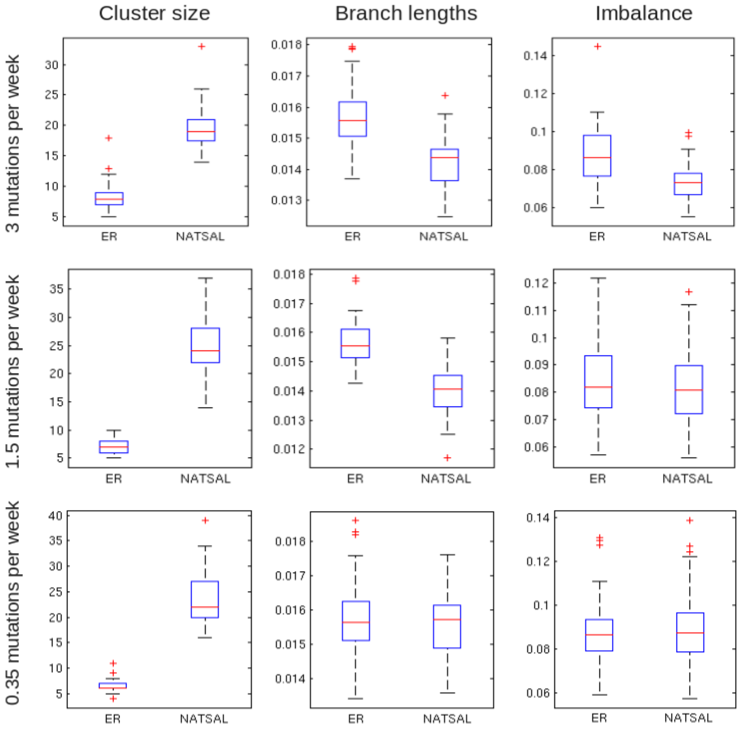

Figure S9: Cluster numbers, branch length and imbalance for the prevalence matched simulations of the main text, as the mutation rate is lowered. The top row are the data shown in the main text. The middle and bottom rows reflect lower mutation rates as shown in the text at the left.

Phylogenetic error

The mutation rate in the main results was sufficiently high that phylogenetic error should not have played a role in shaping the results we reported. However, particularly for bacterial pathogens in situations of rapid transmission, under some circumstances, mutations may not accrue rapidly enough for genealogies to be reliably inferred from phylogenetic data. We therefore explored lower mutation rates, and Figure S9 shows the results. While we have argued that differences in cluster numbers and sizes are highly variable and therefore are not robust detectors of network differences, they are the most robust to phylogenetic error.

Static	ER	NATSAL
number of nodes	50000	50000
number of edges	112296	76672
max degree	16	99
mean degree	2.245920	1.53344
mean clustering	0	0.0017
giant connected component	49456 nodes	29642 nodes
mean shortest path	7.5	6.96

Dynamic	ER	NATSAL
number of nodes	50000	50000
number of edges	17223 (120)	9771 (235)
max degree	6.5 (5)	21.7 (2.8)
mean degree	0.344 (0.002)	0.195 (0.005)
mean clustering	0	0.0001 (0.00003)
giant connected component	63.6 (15)	283 (23)
mean shortest path	9 (1)	5.3 (0.22)

Table S1: Summary of network properties. Results reported for dynamic networks are based on an average of 10 snapshots taken of the networks between 100 and 200 weeks, when relationship dynamics have stabilised. Because they are snapshots and relationships are dynamic, only a few of the existing relationships are present in a given snapshot, so the numbers of edges (at a time) in the dynamic networks are considerably smaller than the numbers of edges in the corresponding static networks. Numbers in parentheses are the standard deviations of reported values over these snapshots.

References

[1] A.M. Johnson, C.H. Mercer, B. Erens, A.J. Copas, S. McManus, K. Wellings, K.A. Fenton, C. Korovessis, W. Macdowall, K. Nanchahal, et al. Sexual behaviour in britain: partnerships, practices, and hiv risk behaviours. *The Lancet*, 358(9296):1835–1842, 2001.
